# Seaweeds in Pig Nutrition

**DOI:** 10.3390/ani9121126

**Published:** 2019-12-12

**Authors:** Carlo Corino, Silvia Clotilde Modina, Alessia Di Giancamillo, Sara Chiapparini, Raffaella Rossi

**Affiliations:** 1 Dipartimento di Medicina Veterinaria, Università degli Studi di Milano, Via dell’Università 6, 26900 Lodi, Italy; carlo.corino@unimi.it (C.C.); alessia.digiancamillo@unimi.it (A.D.G.); 2 Dipartimento di Scienze Veterinarie per la Salute, la Produzione Animale e la Sicurezza Alimentare, Università degli Studi di Milano, Via Celoria 10, 20133 Milan, Italy; silvia.modina@unimi.it (S.C.M.); sara.chiapparini@unimi.it (S.C.)

**Keywords:** seaweeds, dietary supplement, pig

## Abstract

**Simple Summary:**

In pig nutrition, alternative and safe supplements are needed to enhance the pigs’ health and welfare. Natural feed components, such as herbs and plant extracts, are of great importance in animal nutrition, and marine macroalgae can be considered as supplements positively influence animal health parameters. Seaweeds possess several bioactive molecules that are studied for their prebiotic, anti-microbial, antioxidant, anti-inflammatory and immunomodulatory effects. Seaweed benefits are related to their content of sulfated polysaccharides, phlorotannins, diterpenes, omega-3 polyunsaturated fatty acids, minerals and vitamins. This paper reviews the following biological functions of seaweeds and seaweed extracts in pig nutrition: prebiotics, anti-microbial, antioxidant, anti-inflammatory and immunomodulatory effects, promoting intestinal well-being and improving digestibility.

**Abstract:**

Seaweeds are macroalgae, with different sizes, colors and composition. They consist of brown algae, red algae and green algae, which all have a different chemical composition and bioactive molecule content. The polysaccharides, laminarin and fucoidan are commonly present in brown seaweeds, ulvans are found in green seaweeds and, red algae contain a large amount of carrageenans. These bioactive compounds may have several positive effects on health in livestock. In order to reduce the antimicrobials used in livestock, research has recently focused on finding natural and sustainable molecules that boost animal performance and health. The present study thus summarizes research on the dietary integration of seaweeds in swine. In particular the influence on growth performance, nutrients digestibility, prebiotic, antioxidant, anti-inflammatory, and immunomodulatory activities were considered. The review highlights that brown seaweeds seem to be a promising dietary intervention in pigs in order to boost the immune system, antioxidant status and gut health. Data on the use of green seaweeds as a dietary supplementation seems to be lacking at present and merit further investigation.

## 1. Introduction

Marine-derived bioactive compounds are valuable as food and feed ingredients due to their biological activities [1]. The term “algae” includes photosynthetic organisms that are usually divided into microscopic unicellular organisms, identified as microalgae, and multicellular large-size organisms defined as macroalgae or seaweed.

Microalgae usually grow in seawater and freshwater environments and can be prokaryotic, similar to cyanobacteria (*Chloroxybacteria*), or eukaryotic, similar to green algae (*Chlorophyta*). Diatoms (*Bacillariophyceae*), green algae (*Chlorophyceae*) and golden algae (*Chrysophyceae*) are the most abundant but blue-green algae (*Cyanophyceae*) are also defined as microalgae [2]. The bioactive molecules of microalgae are used as food and feed supplements [3]. 

Seaweeds are marine organisms and comprise thousands of species, which are classified on the basis of their pigmentation: brown seaweeds (*Phaeophyceae*), red seaweeds (*Rhodophyceae*) and green seaweeds (*Chlorophyceae*).

There are around 1800 species of brown seaweeds include, only 1% of which are recognized from freshwater and the size range varies from 20 m to 30 cm long. The brown color of these algae is related to the main content of carotenoid fucoxanthin, which masks β-carotene, violaxanthin, diatoxanthin, and chlorophyll. The main polysaccharides are laminarin, fucoidans and alginates, and the cell walls are composed of cellulose and alginic acid [4]. 

Like brown seaweeds, red algae (about 6100 species) are marine, but are able to photosynthesize in deeper water. The size ranges from thin films to filamentous and membranous forms of 1 m. The color results from the presence of the pigments, phycoerythrin and phycocyanin which mask α, β carotene, lutein, zeaxanthin and chlorophyll [5,6]. The main reserves are typically floridean starch and floridoside, and the cells wall are made up of long-chain polysaccharide agars, carrageenans and cellulose [7]. 

There are around 2200 species of green seaweeds. They are of similar size to red seaweeds, only 10% are marine, and their color is related to the presence of chlorophyll. The reserves are composed of starch, and the cells wall are made up of polysaccharide ulvan [8].

In 2015, world production of algae amounted to 30.4 million tons, of which about 96% from aquaculture and only 1 million tons from harvesting of wild stock [9].

Due to their nutritional value and the content of bioactive molecules, seaweeds are often used as food, herbal medicines, dietary supplements, as a source of agar, alginate and carrageenan for several industrial applications, and as a fertilizer [10,11]. 

## 2. Chemical Composition

Several studies have been carried out in order to identify the nutritional composition and secondary metabolites of various seaweed species. In fact, it has been reported that seaweed contains several metabolites, such as the sulfated form of polysaccharides, omega 3 fatty acids, phlorotannins, diterpenes, vitamins and minerals, thus demonstrating positively health effects such as antibacterial, antioxidant, anti-inflammatory functions [12]. The chemical composition of seaweeds was found to vary in relation to their species and genera, harvesting period, and habitat condition (water temperature, light, salinity, nutrients) [13]. 

The chemical composition and mineral content of brown, red and green seaweeds are reported in Table 1. As shown, there is a different nutritional composition range for brown, red and green seaweed, although in the same genus, the values are comparable [14]. 

Of the brown seaweeds, common species such as *Ascophyllum, Laminaria, Saccharina, Macrocystis, Fucus,* and *Sargassum* was considered [15]. Brown seaweed shows a highly variable composition but presented a low protein (7.6–12.6% dry matter, DM) and fat content (0.8–6% DM). The *Fucus* species presents the highest protein content (12.9% DM), followed by *Sargassum* (10% DM), *Laminaria* (9.4% DM) and the *Ascophillum nodosum* (7.4% DM), as observed by Fleurence et al. [16]. The fat content of brown seaweeds is generally lower with an average value of 3.2% DM, and high values are observed in *Fucus* spp. and *Ascophillum nodosum* [17,18].

Red seaweeds contain a higher protein content (16.9% DM) and fat content (8.9% DM) than brown seaweeds [19].

The green seaweed *Ulva lactuca* has a protein content (16.2% DM) compared to red seaweeds and a comparable fat content (1.3% DM) with brown seaweeds [20]. The fat content of the studied seaweeds varies between 0.8 to 8.9% which is a similar range reported for other seaweeds species [13].

All seaweeds are characterized by a higher ash content (19.3–27.8% DM) than those observed in edible plants, in fact they are a considerable rich source of minerals for livestock nutrition [18,19]. 

Seaweeds are rich in potassium, sodium and calcium. Although there is a high variability, in general, the sodium and potassium contents in *Ulva* spp. are lower than those reported for red and brown seaweeds. A higher content of potassium has been observed in *Palmaria palmata, Macrocystis pyrifera*, and *Laminaria* spp. [19]. All seaweeds present higher levels of calcium than phosphorous, and thus may be a possible natural source of calcium in livestock. Seaweeds are also a source of essential trace elements such as iron, manganese, copper, zinc, cobalt, selenium and iodine. In particular, iron is abundant in all the species considered, and the iodine content is higher in brown than in red and green seaweeds (*Laminaria* spp., with a range 833–5100 mg/kg DM), and a higher zinc content has been observed in red and brown than in green seaweed. 

The bioavailability of minerals is related to the fiber content of seaweeds. In addition, the interactions with several polysaccharides, such as alginates and agar or carrageenan, lead to the formation insoluble complexes with minerals, decreasing their bioavailability [21]. The mineral content in the insoluble indigestible fraction residues was higher in brown than in red seaweeds with a range of 150–260 g/kg [22]. Some studies in vitro and in rats have been performed on the bioavailability of minerals [21]. In an in vitro study of 13 seaweed species, only *Palmaria palmata* and *Ulva lactuca* showed higher Fe bioavailability than spinach, although six species had a higher Fe content. The apparent absorption values of Na and K were significantly higher in rats supplemented with *Laminaria* spp., while Mg absorption was not affected. It has also been reported that *Laminaria* spp. is rich in alginates, which probably hampers the bioavailability of Ca. The absorption of inorganic I, which is the predominant form in brown seaweeds, was observed to be moderate (20–70%). Therefore, the low bioavailability may be related to the iodine interaction with other compounds in the seaweed matrix.

The vitamin content showed that seaweeds are a source of water-soluble vitamins (B1, B2, B3 and C) and fat-soluble vitamins (E and provitamins carotenoids, with vitamin A activity). Seasonal effects have a great influence on vitamin content. Most of the red seaweeds, such as *Palmaria palmata* contained a considerable amount of provitamin A and vitamins B1 and B2. The brown seaweeds *Laminaria* spp., *Ascophillum nodosum* and *Fucus* spp. showed a high content of vitamins E and C [23].

The amino acid composition of different seaweeds species is reported in Table 2. Red seaweeds have a higher quality of protein than brown and green seaweeds [24], however there is a considerable difference in the amino acidic content among seaweeds, in relation to the different seasons. It has been reported that seaweeds have a low content of methionine and histidine [25,26]. Leucine was the most abundant amino acid, ranging from 2.43 g/kg DM to 6.63 g/kg DM for *Palmaria palmata*. and *Ascophillum nodosum* respectively, followed by lysine (1.42–7.60 g/kg DM), threonine (1.26–5.17 g/kg DM) and valine (2.25–5.87 g/kg DM). Glutamic and aspartic acids are the most common amino acids found in the non-essential fraction which are responsible for the flavor and taste of seaweeds [27].

The in vitro protein digestibility (IVPD) was mid-range (82–87%) for *Saccharina latissima* and *Palmaria palmata*, and lower (79%) for *Ascophillum nodosum* and *Fucus* spp. The Red seaweed IVPD of red seaweeds had an average value of 85%, while the brown seaweeds had a lower IVPD, with an average value of, 79.7%. A significant inverse correlation between the IVPD and total phenolic content was also observed [28].

Seaweeds also possess several biological activities due to the presence of several bioactive compounds, such as phenolic compounds, carotenoids, tocopherols, polysaccharides, and peptides. Seaweeds are rich in carboxylated and sulfated polysaccharides, such as alginates, ulvans and fucoidans: their composition and content depend on the algae species and of environmental factors such as the season and temperature. The total polysaccharide content (% DM) is 29–67% in green algae (Chlorophyta), 10–59% in red algae (Rhodophita) and 10–75% in brown algae (Phaecophyta). [29]. 

A major component of brown seaweed cell walls is a salt form of alginic acid, alginate with the content ranging from 140–400 g/kg DM [30]. Brown seaweed walls are also rich in fucoidans, which are saccharide units with different degrees of sulphation. Several species contained fucoidan with distinct structural characteristics, and thus the different types of functional components have various biological applications. The fucoidan content varies in relation to seaweed species and season, although the content ranges from 20 to 200 g/kg DM, with the highest value in *Fucus vesiculosus* [17,30,31]. Laminarin, composed of (1,3)-b-D-glucopyranose residues, is the main reserve carbohydrate of brown seaweeds with a content ranging from 0 to 300 g/kg DM. In Laminaria spp. and *Saccharina latissima* a high content of laminarin has been reported, while, *Ascophyllum nodosum* and, *Fucus* spp. presented a low laminarin content [17,30,31]. Laminarin presents prebiotic, immunomodulator and antioxidant activities [32]. 

The cell walls of red seaweeds are mainly composed of sulfated galactan such as carrageenans (content range: 220–770 g/kg DM) and agars (content range: 210–420 g/kg DM). Some red seaweed species contain xylan (in *Palmaria palmata* approximately 350 g/kg DM) and porphyran (average content 480 g/kg DM). The floridean starch is the main carbohydrate reserve with a content ranging from 250 to 420 g/kg DM [17,30,31].

Ulvan is one of the main sulfated polysaccharides from green seaweed cell walls with anticancer, antioxidant, antihyperlipidemic, and anticoagulant activities [33] and a content ranging from 400 to 500 g/kg DM [30]. 

For details and information on the monosaccharide composition of the different polysaccharides mentioned, some excellent reviews are available [34,35].

Considering their valuable source of bioactive molecules, seaweeds have been studied as feed supplements in livestock, particularly in pigs in order to boost growth performance and health [36]. Seaweeds exhibit prebiotics, anti-microbial, antioxidant, anti-inflammatory and immunostimulant properties and obviously effective absorption of dietary nutrients.

## 3. Influence on Growth Performance

Brown seaweeds have a generally positive effect on growth, as presented in Table 3. Some interactions between seaweeds bioactive molecules and dietary components should be probable, but considering the heterogeneity of seaweeds species, the effects on growth performances have to be firstly analyzed in relation to seaweed supplement, and bioactive molecules content. With dietary integration in sows at the end of gestation and during lactation, an increase in average daily gain (ADG) of suckling piglets has been observed (from +11.8 to +32.3% compared to the control group). Most of the studies we reviewed involve the dietary supplementation of brown seaweeds in weaned piglets. In weaned piglets, improvements of ADG are observed. The ADG of piglets fed brown seaweeds is higher than the ADG of piglets fed a control diet with an increase of between +4.6 and +40.8%. 

Draper et al. [56] and Ruiz et al. [57] appear to be the only two authors to report the effects of long–term dietary supplementation with brown seaweed from weaning to slaughter on ADG. In this case, the influence on ADG was limited but statistically significant, and ranged from +1.2 to +3.3%. Bouwhuis et al. [58,59] evaluated the effects of brown seaweed supplementation on pigs’ growth performance after being challenged with *Salmonella Typhimurium*. When the challenge occurred in post–weaning, no significant effect was observed; in pigs with a live weight of 30 kg, the seaweed supplement led to a significant increase in growth (+16%). It is possible that the bioactive compounds of seaweeds are not able to positively modulate the immune system of the post–weaning piglet which is still immature.

Positive effects on growth are related to the improvement in digestibility and overall health conditions of piglets due to the prebiotic effects of seaweed polysaccharides, as described in the following sections. The effects of seaweed dietary supplementation on the improvement in antioxidant status and the decrease in inflammatory condition may contribute to reduce energy and amino acidic expenditure.

## 4. Influence on Digestibility 

Many authors have evaluated the effects of algae supplementation on the digestibility of the diet in pigs, as presented in Table 4. 

All digestibility trials were conducted in weaned piglets, except for the study by Gardiner et al. [72] which investigated male pigs with a 45 kg live weight. The *Ascophyllum nodosum* does not appear to have a significant influence on diet digestibility [42,72]. On the other hand, *Laminaria digitata, Laminaria* spp., *Ecklonia cava* and brown seaweed, titrated in alginates, showed positive effects on the digestibility of nitrogen (N), gross energy (GE), fiber (NDF) and ash in various experiments. Significant improvements from +5.1 and +8% in N digestibility are reported. Also for GE, dietary integration with seaweed improved the digestibility, with an increase of between +3.3 and +10%.

Some authors have also observed that introducing laminarin and fucoidans in the formula increases the digestibility of the fibrous fraction (NDF). The animals fed seaweed showed a higher digestibility of NDF (+39 to +73%) compared to the control group. Finally, ash digestibility presented values that in the seaweed group were 25.9–82.4% higher than in the control. 

The improvement in nutrient digestibility is related to the influence of the seaweed constituents, in particular carbohydrates and antioxidants, on microbiota and on the villous architecture with an increase in absorptive capacity and nutrient transporters [73]. These effects are also related to the trophic effect on the intestinal mucosal cells of volatile fatty acid production (i.e., butyric acid).

## 5. Prebiotic Function

Seaweeds are rich in carboxylated and sulfated polysaccharides, such as alginates, ulvans and fucoidans which all act as prebiotics with positive effects on gut health. According to FAO [74] a prebiotic is a ‘non–viable food component that confers a health benefit on the host associated with the modulation of microbiota’. The health benefit is associated with the stimulated activity/growth of beneficial bacteria and the higher production of short chain fatty acids (SCFAs) with direct impact on gut health and also an immunomodulatory effect, as reported below. 

Several papers have analyzed the prebiotic effects of algae [5,29,73,75,76,77,78,79]. In swine, 24 studies have been published in the last 10 years on the effects of supplementation with brown seaweeds, or their extracts, on gut health: *Ascophyllum nodosum* [42,60,72], *Ecklonia cava* [63], *Laminaria digitata* [64,80,81], *Laminaria hyperborea* [82,83], *Laminaria digitata* and *Laminaria hyperborea* association [84], *Laminaria* spp. [58,59,65,67,69,70,71,85,86,87,88,89,90]. Brown seaweeds titrated in alginic acid polysaccharides have also been studied [63]. Most of the studies were carried out on weaned piglets (14 trials), considering that the weaning phase is a critical period with a high incidence of enteric pathologies. Some studies were carried out on growing pigs ranging between 14 and 65 kg LW, and some others on gestating and lactating sows.

In general the compounds present in the brown seaweeds (in 20 trials the supplement was titrated in laminarin and/or fucoidans) stimulated the growth of *Lactobacilli* [61,64,65,70,71,72,81,82,83,84], and reduced the enterobacteria population or *Escherichia coli* [42,58,61,63,64,69,71,72,83,84,85,87]. Brown seaweed supplements supported the growth of *Bifidobacteria* species in the ileum in piglets [61,80,81]. Gut health is modulated by laminarin and/or fucoidans, with the microbial production of short–chain fatty acids (SCFAs), in particular butyrate [81,84,86]. Glucose are the main energy source for small intestinal epithelial cells, and SCFAs are the main energy source for caecum and colon cells, stimulating cell growth [91]. Several studies have reported that brown seaweeds have a positive influence on gut morphology [63,87,88,92]. Supplementation with *Ecklonia cava* (0.05 and 0.15% of dietary inclusion), linearly improved villi height in the ileum [63]. In weaning piglets, maternal dietary supplementation with laminarin and fucoidans (1 and 0.8 g/day) after 83 days of gestation and during lactation increased villi height in the jejunum and ileum (+43 and +88% respectively) [87]. According to Heim et al. [88], maternal dietary treatment with fucoidans (0.8 g/day) had no influence on the small intestine morphology, while laminarin increased the villus height in the ileum (+13%) at day 8 post–weaning. In vitro and in vivo experiments carried out by Dierick et al. [42] revealed that native seaweeds *Ascophyllum nodosum* suppressed in vitro the gut flora counts and metabolic activity (production of organic acids), while in vivo, a significant better *lactobacilli/E. coli* ratio was found in the small intestine. Michiels et al. [60] on the other hand observed no significant effects on gut health with the use of the same seaweed in weaned piglets, most probably due to the already high digestible basal diet, including lactose. To probiotic activity algae associate bacteriostatic and antibacterial activities recently reviewed by Perez et al. [93]. In particular potential applications in aquaculture [94] and in the pharmaceutical and food industry [95], have been evaluated.

## 6. Antibacterial Function

In addition to the probiotic action that positively modulates the intestinal microbiota, the seaweeds and their extracts show a specific anti-bacterial and / or bacteriostatic action [8,96,97]. Phlorotannins, fatty acids, peptides, terpenes, polysaccharides and sulphated polysaccharides, and several other bioactive compounds have been reported as bacterial inhibitors (Table 5). A very interesting action of seaweed extracts is the effectiveness against methicillin resistant *Staphylococcus aureus* and vancomycin–resistant Enterococcus faecium [98].

Antibacterial activity is expressed across multiple mechanisms: inhibition of oxidative phosphorylation and link with compounds in the bacterial cell wall and increased permeability of the cytoplasmic membrane causing cell lysis [97]. At the same time some seaweed compounds, in particular polysaccharides, contribute significantly to the health and well-being of the animals by enhancing the in vivo immune response. 

The variability of bioactive compounds in seaweeds influences the lab techniques used to obtain antimicrobials ranging from traditional extraction techniques, solid–liquid extraction or liquid–liquid extraction, to the most modern process of supercritical fluid extraction using CO2 ultrasonically-assisted extraction [93]

The potential applications in aquaculture [94] and in the pharmaceutical and food industry [95], have been evaluated, while studies on food producing animals, and in pigs in particular, are limited. Berri et al. [112] evaluated the effect of marine-sulfated polysaccharide extract from the green macroalga Ulva armoricana against seven bacterial strains found in pigs (Table 6).

## 7. Influence on Antioxidant Function

Seaweeds have antioxidant properties due to the presence of phenols, carotenoid fucoxanthin, tannins and phlorotannins, polysaccharides (fucoidans and laminarans in brown seaweeds; ulvans in green seaweeds and carrageenans, porphyrin and agar in red seaweeds) [113]. The highest concentrations of phenols and phlorotannins have been observed in brown seaweed, up to 12–14% DM in *Ascophhyllum nodosum, Fucus* spp. and *Sargassum* spp. [114]. In green and red seaweeds concentrations lower than 1% have been reported [114,115,116].

The carotenoid fucoxanthin has only been detected in brown seaweeds with concentrations of up to 5.000 mg/kg [117,118,119]. Tocopherols are present in all seaweed, with variables concentrations. In brown seaweed higher values have been reported for *Fucus* spp. and *Ascophyllum nodosum* (up to 600 mg/kg DM) [120,121] than in *Laminaria* spp. [122]. Lower concentrations were observed in red seaweeds [122,123], and green seaweeds showed up to 1070 mg/kg DM in *Ulva* spp. [47]. 

The effects of dietary seaweeds are reported on serum and plasma antioxidant status, duodenum, jejunum and ileum antioxidant markers and *Longissimus dorsi* muscle oxidative stability (Table 7).

At the blood level, dietary supplementation with *Laminaria* spp. extract (laminarin 0.18 g/kg and fucoidans 0.33 g/kg) or brown seaweed (alginates 100 mg/kg) has a strong antioxidant effect in growing pigs [124] and weaned piglets [61,62]. The total antioxidant capacity (TAS), superoxide dismutase (SOD), glutathione (GSH) and catalase activities increased from +14 to +37% with respect to the control group. At the same time, there was a reduction in lipid oxidation with lower values of malondialdehyde (MDA) ranging from 10% to 26% than in the controls. Other authors reported the non-significant effects on serum MDA, using dietary *Ascophyllum nodosum* [60] and brown seaweeds [62].

Wan et al. [92] observed a significant reduction in the MDA concentration in duodenum, jejunum and ileum of between –35 and −40% and an increase in catalase activity. Finally, some studies have evaluated the oxidative stability of pork during storage time by evaluating thiobarbituric acid-reactive substances (TBARS). The reduction in TBARS concentration for long refrigerated storage times of 14 d, ranged between −21% and −60%. The administration of an extract of *Laminaria digitata* in liquid form instead of spray–dried appears to affect the antioxidant potential of the extract. The liquid form better exploits the antioxidant potential of the extract, with reductions in TBARS production of –47% compared to –29% with the spray-dried form [125].

## 8. Anti-inflammatory Function

Many studies have evaluated the anti–inflammatory activity of brown seaweeds, in particular *Laminaria digitata, Laminaria hyperborea* and *Laminaria* spp., usually titrated in laminarin and fucoidans [58,59,61,71,80,85,87,88,90,92]. In addition, non-specified brown seaweeds titrated in alginic acid have been evaluated by Wan et al. [61,92]. Of these authors, 8/10 showed anti-inflammatory effects. Walsh et al. [71] reported a lower expression of pro-inflammatory cytokines in the colon of piglets after dietary supplementation with laminarin, but not with fucoidans. 

Dietary treatment of gestating and lactating sows with laminarin (1 g/d) and fucoidans (0.8 g/d) reduced the ileal gene expression of IL-6, IL-8, IL-10 of piglets at weaning [87]. Similarly, in piglets born from sows fed diets supplemented with laminarin from 109 d gestation and during lactation, a reduction on the colon IL-6 concentration at weaning and of ileal IL-8 concentration eight days post weaning were observed [88].

In a 28-day trial, Bouwhuis et al. [59] observed the effects of a diet supplemented with laminarin 0.18 g/kg and fucoidans 0.34 g/kg, in 30 kg LW female pigs. After an 11-day adaptation period, pigs were orally challenged with *Salmonella Typhimurium*. Dietary treatment reduced colon cytokine expression (IL-6, IL-18, IL-22 and TNF-α) 17 days post challenge, thus revealing an anti-inflammatory effect. In addition, in 18 kg LW pigs, laminarin from *Laminaria digitata* dietary supplementation (0.6 g/kg) significantly increased gut mucin gene expression (MUC2 and MUC4) from 20% to 33% with a protective effect on epithelial cells [127]. McDonnel et al. [90] observed an increase of 16% in mucin gene expression (MUC2) in the ileal in female pigs fed diets supplemented with *Laminaria* spp. (0.18 g of laminarin and 0.34 g of fucoidan per kg of feed). A study using an in vitro system of porcine intestinal epithelial cells showed that ulvans from *Ulva armoricana*, a green seaweed, upregulated the gene expression of cytokines such as IL1α, IL1β, L6, IL8, TNFα [112,128].

## 9. Immunomodulatory Function

Many studies have evaluated the immunomodulatory activity of seaweeds [61,63,85,129,130,131]. In addition to an anti-inflammatory action, laminarin also has an immunomodulatory function. Leonard et al. [85] reported that the dietary supplementation of sows from 109 days of gestation until weaning with *Laminaria* spp. extract (1 g laminarin and 0.8 g fucoidans/day) increased immunoglobulin G (IgG) and immunoglobulin A (IgA) in sow colostrum by 19% to 25%, respectively. Consequently, an increase in piglet serum IgG was observed (10%). 

In another study, the effects were evaluated of sow supplementation with 30 g/day of an extract of *Ascophyllum nodosum* and *Fucus* from the 85th day of gestation until weaning on liver and lymphoid organs of piglets. The relative population of CD4 + CD8 + T cells was higher in piglets from treated sows in the thymus, spleen, mesenteric node, liver and in peripheral blood, thus suggesting an important effect of maternal diet on the immune status of 40-day-old piglets [130]. 

The immunomodulatory effect of green seaweed extract (*Ulva armoricana*) was evaluated in sows by Bussy et al. [131]. Different levels of inclusion were tested: 2, 8 and 16 g/day during two periods of three days: 34 days before farrowing, before the last vaccine booster against *Bordetella bronchiseptica*, and one week before farrowing. The higher dietary level increased *anti-Bordetella* IgG in sow’s blood and colostrum, while with the middle dietary integration, the authors observed an increase in milk IgA. Wan et al. [61] and Choi et al. [63] evaluated the immunomodulatory effects of seaweed fed to weaned piglets. Alginic acid oligosaccharides from brown seaweed increased IgG and IgA concentrations in piglet serum by 20% and 53 %, respectively after 21 days of treatment [61]. No immunomodulatory effect was observed in weaned piglets fed a diet supplemented with different *Ecklonia cava* concentrations [63]. According to the authors, the result may be the consequence of the low dosage and consequently the low content of fucoidan in the diets (0.056 – 0.112 – 0.168 g/kg, respectively). 

In growing pigs (29 kg LW), 0.8% seaweed enhanced the immune function. Pigs were sensitized with the subcutaneous inoculum of sheep red blood cells at days 42 and 49. Seaweed increased the saliva IgA production five times more than the control after 56 days [129]. The concentration of antigen-specific IgG in peripheral blood was higher in the seaweed group, but not significantly due to the high standard deviation. 

## 10. Potential Toxicity 

The use of seaweed in swine nutrition may have the following limitations: mineral elements and potentially toxic mineral content. Generally, seaweeds are introduced in pig diets as ingredients/raw materials in a low percentage, thus there is no risk of potential toxicity in this case. In terms of microelements, the first limiting factor is the iodine content which, as reported in Table 1, can reach particularly high concentrations in brown seaweeds. According to NRC [132], tolerance levels in growing pigs and sows are 400 and 1500–2500 mg/kg DM for iodine from iodine salt, respectively. Of the potentially toxic minerals, the first limiting element is arsenic (As) which is found in high concentrations in brown seaweed (Table 8). However, a low content of the most toxic form of arsenic, inorganic arsenic, has been observed in seaweeds. The arsenic content of green seaweed is below the maximum level of 40 mg/kg feed (12% moisture content) set by European feed legislation (Commission Directive 2002/32/EC and amendments).

## 11. Conclusions 

The biological activities of brown seaweeds could be used to improve the health and welfare of pigs. The prebiotic effects and the antimicrobial activities of laminarin and fucoidans may have beneficial effects in the prevention of gastrointestinal diseases and to enhance diet digestibility in post–weaning piglets. Laminarin also has an anti-inflammatory activity, which reduces the pro–inflammatory cytokine response. The seaweed content of antioxidant molecules enhances the antioxidant status and meat oxidative stability. Dietary supplementation with brown seaweed may positively affect the immune system, enhancing immunoglobulin production and modulating cytokine production. In conclusion, brown seaweeds seem to be a promising dietary intervention in pigs in order to enhance the immune system, antioxidant status and gut health. Data on the dietary supplementation with green seaweeds in pigs seem to be lacking at present and merit further investigations.

## Figures and Tables

**Table 1:** Chemical composition of brown, red and green seaweeds (on DM basis) ^1^.

Seaweeds	BROWN						RED	GREEN
	*Laminaria* spp. *	*Ascophillum* *nodosum*	*Sargassum* spp. ⁑	*Fucus* spp. #	*Saccharina* *latissima*	*Macrocystis* *pyrifera*	*Palmaria* *palmata*	*Ulva* *lactuca*
**Crude protein %**	9.4 (5.3–16.1)	7.4 (4.9–8.7)	10 (8.5–13.6)	12.6 (12.2–12.9)	7.6 (7.1–8.1)	8.3 (8–10)	21.9 (15.1–31.4)	16.2 (7.06–23.1)
**Ether extract %**	1.1 (0.8–2.4)	5.3 (3.9–8.6)	0.8 (0.5–1.2)	6.1 (3.7–8.4)	5.5	1.8 (0.5–3.9)	8.9 (4.9–12.9)	1.3 (0.25–1.64)
**Crude Fiber %**	11.6 (6.6–16.6)	5.5 (5.4–5.5)	18.2 (6.4–38)	10.7 (5.4–16)	23 (6.6–40)	33.4 (5.5–50)	1.5 (1.49–1.50)	9.6 (6.9–12.3)
**Ash %**	27.8 (19.6–31.5)	24.8 (21.1–30.9)	27.6 (19.4–35.9)	21.6 (20.7–22.5)	22.5 (13.3–31.7)	25.8 (20–35)	19.3 (9–24.5)	25.7 (21.3–26.2)
**Gross energy MJ/kg**	12.7 (12.5–13)	14.1	9.1	15.7 (15.5–16)	11.1	9	16.9	15.2 (14.7–15.7)
**Ca g/kg**	10 (8–12.55)	16.4 (9.8–20)	14.7 (3.8–27.2)	9.9 (8.9–12.8)	9.8 (9.6–10)	14.1 (11.6–16.6)	2.6 (1–4.2)	12.6 (6.1–29.2)
**P g/kg**	2.2 (1.2–3)	1	1.7 (1–2.2)	1.9 (1.4–2.3)	2.7 (2.2–3.1)	2.9 (2.6–3.2)	4 (3–5)	2.1 (1.3–2.7)
**K g/kg**	54 (48.6–59.5)	28.5 (20–37.7)	46.2	22.9 (0.4–36.1)	52.5	67.5 (44.8–112.3)	37.1 (27–47.2)	14.4 (1.5–22.1)
**Na g/kg**	23.9 (22.5–25.3)	37.5 (25–45.7)	–	24.2 (0.2–45.8)	33	36.9 (17.1-56.7)	7.2 (3.3–11)	13.9 (2.9–20.2)
**Mg g/kg**	6.3( 5.5–7.2)	6.8 (1–8.6)	6.4 (4–7.7)	7.5 (7–8.33)	6.3 (5.1–7.4)	39 (16.2–61.8)	2.3	13 (1.9–20.5)
**Mn, mg/kg**	7.1 (3.1–11)	17.8 (12–25)	88.3 (26.7–214)	104.7 (8.2–177.8)	8.2 (3.9–12.4)	11	71.6 (11–168)	38.7 (10.1–122)
**Zn mg/kg**	22.6 11–31.5)	116.8 (30.3–181)	79.3 (12–214)	118.1 (45.3–275.3)	35.4 (29.2–41.55)	12	65.1 (23.6–143)	29 (16.1–45)
**Cu mg/kg**	2.4 (1.2–5.9)	17.8 (4.2–28)	6. (2.3–7)	9.3 (2–23.5)	4.5 (1.1–7.9)	2	11.1 (3.8–24)	8.5 (3.3–12)
**Fe mg/kg**	107.3 (58–179)	157.8 (122–241)	2678 (307–7291)	351.9 (189–559)	529 (30–1028)	117	202.5 (139–315)	462.2 (105–1481)
**I mg/kg**	2991.7 (833–5100)	777	399.5 (216–583)	376 (232–677)	1448.5 (957–1940)	–	278	56.7
**Se mg/kg**	0.6 (0.29–0.93)	0.5	1.2 (1.1–1.4)	0.8 (0.2–1.2)	1.1 (0.9–1.3)	–	0.1	1.2 (0.4–1.9)
**Co mg/kg**	0.1 (0.08–0.11)	0.6	0.4 (0.36–0.47)	1.1 (0.8–1.4)	0.4	–	0.03	0.5 (0.3–0.6)
**Vitamin E mg/kg**	672 (3–2000)	230 (80–500)	10	164 (100–356)	1.6	928	69.6 (22–152)	12.95 (2.8–35)
Vitamin A mg/kg **	154.6 (22–299)	57 (35–80)	51	17.8 (7–28.6)	0.42	12.39	142.9 (15.2–270)	3.5 (0.1–7)
**Vitamin C mg/kg**	632 (355–910)	860 (81–1650)	560	383 (141–770)	11.5 (3.5–18.8)	–	78.3 (0.7–156)	141.5 (42–241)
**Vitamin B_1_ mg/kg**	7.45 (2.4–12.5)	14(1–27)	4	0.2	0.72 (0.5–0.94)	–	18 (0.7–40)	40
**Vitamin B_2_ mg/kg**	4.9 (1.4–8.5)	7.5 (5–10)	65	0.35	1.7 (1.4–2.1)	–	16 (4.3–19)	5.1 (5–5.3)
**Vitamin B_3_ mg/kg**	314 (158–612)	15	20	–	–	–	45 (10.1–83)	–
**References**	[13,17,19,21,37,38,39,40,41]	[17,18,21,24,28,37,42,43,44,45]	[17,19,24,37,45]	[18,19,21,23,28,37,40]	[19,21,23,28,38]	[17,46,47,48,49]	[17,23,28,37,50]	[17,19,21,23,37,44,47,51]

^1^ data are reported as mean values and ranges. * values from *Laminaria digitata* and *hyperarborea*. ⁑ values from *Sargassum Patens*, *hemifhyllum, henslowianum*. # Values from *Fucus vesciculosus, giuryi, serratus, spiralys.* ** as provitamin carotenoids.

**Table 2:** Aminoacid profile of brown, red and green seaweeds (mg/g DM) ^1^.

Seaweeds	BROWN						RED	GREEN
	*Laminaria digitata*	*Ascophillum nodosum*	*Sargassum* spp. ⁑	*Fucus* spp. #	*Saccharina* *latissima*	*Macrocystis pyrifera*	*Palmaria* *palmata*	*Ulva* *lactuca*
**Lysine**	4.41 (4.1–4.8)	4.77 (4.3–5.4)	3.57 (2.8–4.3)	7.6 (6.7–8.2)	4.05 (4–4.1)	6.63 (5.2–7.5)	1.42 (1.2–1.65)	2.09 (0.5–1.9)
**Histidine**	1.82 (1.3–2.4)	1.63 (1.4–1.9)	0.82 (0.6–1)	1.33 (0.4–2)	2.2 (1.2–3.2)	2.33 (2–2.9)	0.4 (0.3–0.5)	0.83 (0.1–2.0)
**Isoleucine**	2.91 (2.6–3.2)	3.76 (3.1–4.3)	2.70 (1.9–3.5)	3.70 (0.9–6)	3.1 (3–3.1)	4.47 (3.2–5.6)	1.31 (0.7–1.9)	1.5 (0.4–5.2)
**Leucine**	4.93 (4.4–5.4)	6.63 (5.3–7.5)	5.11 (4.4–5.8)	6.42 (1.6–10.5)	5.06 (4.2–5.9)	7.53 (5.5–9.2)	2.42 (1.3–3.6)	2.9 (0.7–6.6)
**Arginine**	3.2 (2.9–3.4)	5.06 (4.2–6.0)	1.6 (1–3–1.9)	3.24 (1.1–4.6)	4.0 (3.9–4.1)	4.87 (3.5–6.1)	1.9 (1.2–2.6)	2.12 (0.5–1.3)
**Methionine**	1.5 (1.4–1.5)	1.91 (1.3–2.5)	0.69 (0.68–0.7)	0.81 (0.2–1.8)	2 (1.9–2.1)	2.18 (1.6–2.6)	0.91 (0.5–2.3)	1.21 (0.2–1.8)
**Phenylalanine**	3.24 (2.8–3.6)	4.23 (3.2–5.0)	2.98 (2.2–3.7)	3.56 (0.9–5.2)	3.82	5.42 (4.1–6.2)	1.7 (0.8–2.6)	2.4 (0.2–3.6)
**Threonine**	3.68 (3.4–3.9)	4.6 (3.6–5.4)	3.9 (3.3–4.5)	3.09 (1.2–5.1)	4.3 (4.2–4.4)	5.17 (3.6–6.7)	1.26 (0.7–2.8)	2.17 (0.5–3.8)
**Tryptophan**	1.74 (1.72–1.76)	–	–	1.22 (0.5–1.9)	–	–	0.4 (0.2–0.6)	0.51 (0.4–0.6)
**Valine**	5.38 (4.7–6.0)	4.76 (4.1–5.5)	3.84 (2.9–4.8)	4.65 (1.1–8.0)	4.1 (3.7–4.5)	5.87 (4.3–7.1)	2.25 (1.1–3.4)	2.03 (0.9–4.4)
**Tyrosine**	1.74 (1.7–1.8)	2.05 (0.9–3.2)	2.35 (1.8–2.9)	2.26 (1.9–2.6)	–	2.8 (2.1–3.5)	1.15 (0.6–1.7)	1.03 (0.5–1.4)
**Alanine**	6.68 (4.5–8.8)	5.95 (5.4–6.5)	4.23 (3.3–5.1)	1.58 (1.5–1.6)	6.8 (5.0–8.5)	4.81 (4.5–5.0)	2.23 (1.1–3.3)	3.22 (0.7–5.9)
**Glutamine**	7.3 (4.6–9.9)	14.5 (1.4–11.6)	19.5 (18.9–20.1)	20.1 (19.6–20.3)	10.5 (10.6–10.4)	14.1 (9.7–18.3)	16.6 (11.0–18.7)	13.2 (11.5–14.8)
**Asparagine**	6 (3.9–8.1)	8.4 (8.3–8.5)	12 (11.5–12–5)	12.8 (10.9–16.7)	9.4 (8.8–9.8)	10.8 (8.3–13.3)	11.3 (9.9–14.3)	10.4 (7.9–12.2)
**References**	[41,52]	[17,18]	[14]	[18,53]	[52,54]	[48,49,55]	[25,28]	[17,18,47]

^1^ data are reported as mean values. ⁑ values from *Sargassum Patens* and *hemifhyllum*. # Values from *Fucus vesciculosus* and *serratus.*

**Table 3:** Effect of seaweed supplement on average daily gain (ADG) in pigs.

Algae Supplement	Dose	Animal	Control	Supplemented	Diff. %	Ref.
*A. nodosum*	Dried seaweed 2.5–5–10 g/kg	Weaning to 28 d	0.220	0.209	−5.0	[60]
				0.198	−10.0	
				0.213	−3.18	
*A. nodosum*	Dried seaweed 10–20 g/kg	Weaning to 11 d	0.027	0.054	+100	[42]
				0.040	+48.14	
*Brown seaweed*	Alginic acid oligosaccharides (50–100–200 mg/kg)	Weaning to 14 d	0.216	0.248 (50)	+14.81	[61]
				0.304 * (100)	+40.78	
				0.301 * (200)	+39.35	
*Brown seaweed*	Alginates oligosaccharides (100 mg/kg)	Weaning to 21 d	0.441	0.516	+17.01	[62]
*Ecklonia cava*	FUC = 0.05 – 0.10 – 0.156 g/kg	Weaning to 28 d	0.344	0.347	+0.87	[63]
				0.368 *	+6.98	
				0.360 *	+4.65	
*Laminaria digitata*	LAM + FUC (0.314 –0.250 g/kg) – lactose 15 or 25%)	Weaning to 25 d	0.275 0.287	0.293 (15% lact.)	+6.55	[64]
				0.350 ** (25% lact.)	+21.95	
*Laminaria* spp.	LAM (1 g/day)—sows, 109 d until weaning at 20 d	20 d lactation Weaning to 26 d Challenge Salmonella Typhimurium at 10 d post weaning	0.340	0.450 **	+32.35	[58]
	LAM (0.3 g/kg)—piglets		0.410	0.370	−16.13	
*Laminaria* spp.	LAM + FUC (0.18 + 0.34 g/kg)	30.9 kg pigs for 28 d Challenge Salmonella Typhimurium at 10d	0.620	0.720 ***	+16.13	[59]
*Laminaria* spp.	LAM (0.112 g/kg) ^y^ FUC (0.089 g/kg) ^z^	Weaning to 25 d	0.281	0.322 **	+14.59	[65]
*Laminaria* spp.	LAM + FUC (1 g + 0.8 g day) − sows LAM + FUC (0.3 + 0.24 g/kg) − piglets	Weaning to 126 d	0.760	0.850 **(lactation effect)	+11.84	[56]
			0.800	0.810 (weaning effect)	+1.23	
*Laminaria* spp.	Extract (1–2–4 g/kg) ^x^ LAM = 0.11–0.22–0.44 FUC = 0.09–0.18–0.36	Weaning to 21 d	0.249	0.274 ***(1 g/kg)	+10.04	[66]
				0.313 *** (2 g/kg)	+25.70	
				0.303 ***(4 g/kg)	+21.69	
*Laminaria* spp.	LAM (0.30 g/kg)	Weaning to 32 d	0.280	0.353 *	+26.07	[67]
*Laminaria* spp.	LAM + FUC (0.30 + 0.24 g/kg)	Weaning to 40 d	0.356	0.374	+5.06	[68]
*Laminaria* spp.	LAM (0.3 g/kg) FUC (0.36 g/kg) LAM + FUC (0.3 + 0.36 g/kg)	Weaning to 21 d	0.288	0.319 * LAM 0.3	+10.7	[69]
				0.302 FUC 0.36	+4.86	
				0.328 LAM + FUC	+13.89	
*Laminaria* spp.	LAM + FUC (0.30 + 0.24 g/kg) ^k^	Weaning to 21 d 21–40 d	0.235	0.239	+1.70	[70]
			0.489	0.523	+6.25	
*Laminaria* spp.	LAM (0.15–0.30 g/kg) FUC (0.24 g/kg) LAM + FUC (0.15 + 0.24 and 0.30 + 0.24 g/kg)	Weaning to 35 d	0.340	0.351 FUC 0.24	+3.24	[71]
				0.334 LAM 0.15	–1.76	
				0.347 FUC 0.24 LAM 0.15	+2.06	
				0.390* LAM 300	+14.71	
				0.358 FUC 0.24 LAM 0.3	+5.29	
OceanFeedSwine	Seaweeed extract (5 g/kg)	21 to 56 d	0.401	0.380	−5.24	[57]
		56–160 d	0.798	0.824 *	+3.26	

Means marked with *, **, *** showed a significant effect of supplement for *p* < 0.05, *p* < 0.01 and *p* < 0.001 respectively; LAM, laminarin; FUC, fucoidan; ^y^ 990 g/kg Laminarin; ^z^ 720 g/kg Fucoidan; ^x^ 112 g/kg Laminarin, 89 g/kg Fucoidan and 799 g/kg ash; ^K^ 455 g/kg Laminarin and 360 g/kg Fucoidan.

**Table 4:** Influence of seaweed on digestibility in swine.

Algae Supplement	Dose g/kg	Animal	Effects on Digestibility	Treatment vs. Control, %	Ref.
*A. nodosum*	Dried intact (2.5 g/kg)	Male Pigs, 45 kg LW	NS	–	[73]
*A. nodosum*	Dried intact (10–20 g/kg)	Weaned piglets (35 d age)	NS	–	[42]
Brown seaweed	Alginates olisaccharides (100 mg/kg)	Weaned piglets, 6.2 kg LW	Improved digestibility of		[62]
			N, fat, ash GE	+6.7% +10.8% +25.9% +4.0%	
*Ecklonia cava*	Seaweed (0.5–1—1.5 g/kg) ^s^	Weaned piglets 7.8 kg LW	Improved digestibility of GE	+3.3% (1g/kg)	[63]
*Laminaria digitata*	LAM + FUC (0.314–0.250 g/kg)	Weaned piglets, 7.2 kg LW	Improved digestibility of		[64]
			OM, N, NDF, GE	+4.5% +7.3% +73.3% +5.9%	
*Laminaria* spp.	Extract (1–2–4 g/kg) ^x^	Weaned piglets (24 d age)	NS		[66]
*Laminaria* spp.	Seaweed extract LAM (0.112 g/kg) ^y^ FUC (0.089 g/kg) ^z^	Weaned piglets, (24 d age)	Improved digestibility of		[65]
			N, GE	+6.7% +5.2%	
*Laminaria* spp.	LAM + FUC (0.30 + 0.24 g/kg)	Weaned piglets (22 d age)	Improved digestibility of		[68]
			DM, N, NDF	+8.8% +8.9% +57.5%	
*Laminaria* spp.	LAM (0,15–0,30 g/kg) FUC (0,24 g/kg) LAM + FUC (0,15 + 0.24 and 0.30 + 0.24 g/kg)	Weaned piglets (24 d age)	improved digestibility of		[71]
			DM, LAM and LAM + FUC OM, LAM and LAM + FUC N, LAM NDF, LAM and LAM + FUC GE, LAM and LAM + FUC	+7.0% – +4.5% +5.9% – +3.5% +5.1% 54.5% – 39.7% +7.3% – +4.3%	
*Laminaria* spp.	LAM (0.30 g/kg) FUC (0.24 g/kg) LAM + FUC (0.30 + 0.24 g/kg)	Weaned piglets (24 d age)	Improved digestibility of		[67]
			DM, LAM and LAM + FUC N, LAM Ash, LAM and LAM + FUC GE, LAM and LAM + FUC	+7.9% – +4.5% +6.6% 58.0% – 42.6% +8.5% – +4.3%	
*Laminaria* spp.	Extract (0.66 g/kg) ^k^	Weaned piglets (24 d age)	Improved digestibility of		[70]
			OM, N, Ash, NDF, GE	+8.8% +8.9% +82.4% +57.5% +10.9%	

^x^ 112 g/kg Laminarin, 89 g/kg Fucoidan and 799 g/kg ash.; ^k^ 455 g/kg Laminarin and 360 g/kg Fucoidan; ^s^ 112 g/kg Fucoidan; ^z^ 720 g/kg Fucoidan; ^y^ 990 g/kg Laminarin; FUC, Fucoidan; LAM, laminarin; LW, live weight; DM, dry matter; GE, gross energy; N, nitrogen; NDF neutral detergent fiber; OM, organic matter.

**Table 5:** Antibacterial activity of seaweeds.

Strain	Seaweed	Functional Group	Seaweed	Ref.
*Campylobacter jejuni*	*Delisea pulchra*	Halogenated furanone	Red	[99]
*Enterococcus faecium* vancomycin-resistant	*Callophycus serratus*	Diterpene-benzoate	Red	[98]
*Escherichia coli*	*Ascophyllum nodosum* and *Laminaria hyperborea*	Laminarin	Brown	[100]
	*Sphaerococcus coronopifolius*	Sphaerane bromoditerpenes	Red	[101]
	*Pterocladia capillacea*	Water-extracted polysaccharides	Red	[102]
	*Sargassum swartzii*	Sulphated polysaccharides	Red	[103]
	*Delisea pulchra*	Halogenated furanone	Red	[104]
*Listeria monocytogenes*	*Ascophyllum nodosum* and *Laminaria hyperborea*	Laminarin	Brown	[100]
*Pseudomonas aeruginosa*	*Solieria filiformis*	Lectin	Red	[105]
	*Sphaerococcus coronopifolius*	Sphaerane bromoditerpenes	Red	[101]
	*Delisea pulchra*	Halogenated furanone	Red	[106,107]
*Propionibacterium*	*Eisenia bicyclis*	Phlorofucofuroeckol	Brown	[108,109]
*Salmonella typhimurium*	*Ascophyllum nodosum* and *Laminaria hyperborea*	Laminarin	Brown	[100]
*Staphylococcus aureus*	*Eisenia bicyclis*	Phlorofucofuroeckol	Brown	[110]
	*Ascophyllum nodosum* and *Laminaria hyperborea*	Laminarin	Brown	[100]
	*Pterocladia capillacea*	Water–extracted polysaccharides	Red	[102]
*Staphylococcus aureus* methicillin resistant	*Saccharina longicruris*	Extracted peptides (>10 kDa)	Brown	[111]
	*Sphaerococcus coronopifolius*	Sphaerane bromoditerpenes	Red	[101]
	*Callophycus serratus*	Diterpene-benzoate	Red	[98]

**Table 6:** Minimum inhibitory concentration (MIC) of marine–sulfated polysaccharides extract from *Ulva armoricana* [112].

Strain	MIC (mg/mL)
*Pasteurella multocida*	1.56
*Pasteurella multocida* subsp. *multocida*	3.125
*Streptococcus suis*	6.25
*Trueperella pyogenes*	50
*Bordetella bronchiseptica*	50
*Escherichia coli K85*	>50
*Escherichia coli K88 (F4)*	>50

**Table 7:** Effects of seaweed on antioxidant capacity in pigs.

Algae Supplement	Dose g/kg or g/day	Animal	Antioxidant Effects	Treatment vs. Control, %	Ref.
*A. nodosum*	Dried seaweed 5–10 g/kg	Weaned piglets, 6.59 kg LW	Plasma TBARS ^a^, FRAP ^b^, α-tocopherol	NS	[60]
Brown seaweed	Alginic acid olisaccharides (100 mg/kg)	Weaned piglets, 7.8 kg LW	Serum		[61]
			T-AOC ^e^ SOD ^f^ CAT ^g^ MDA ^h^	+14% +20% +37% −26%	
Brown seaweed	Alginates olisaccharides (100 mg/kg)	Weaned piglets, 6.2 kg LW	Serum		[62]
			T-AOC CAT GSH^i^ MDA	+21% +28% +28% −10% NS	
Brown seaweed	Alginates olisaccharides (100 mg/kg)	Weaned piglets, 6.2 kg LW	Duodenum		[92]
			T-AOC	+45%	
			MDA	−40%	
			Jejunum		
			T-AOC	+39%	
			CAT	+22%	
			MDA	−36%	
			Ileum		
			T-AOC	+58%	
			CAT	+72%	
			MDA	–35%	
*Laminaria digitata*	Wet (W) or spray dried (SD) seaweed LAM + FUC (0.5+0.4 g/kg)	Pigs, 14.5 kg LW	Plasma TAS ^c^ LD muscle TBARS (refrig. storage 14 d)	NS −29% SD −47% W	[125]
*Laminaria digitata*	LAM + FUC (0.45 or 0.9 g/kg) 3 or 6 weeks pre slaughter	Pigs, 82 kg LW	Plasma TAS LD muscle TBARS (refrig. storage 14 d) 0.45 for 3 weeks 0.90 for 3 weeks	NS −57% −60%	[126]
*Laminaria* spp.	LAM + FUC (0.18 + 0.33 g/kg)	Pigs, 71 kg LW	Serum DPPH ^d^ LD muscle TBARS (refrig. storage 14 d)	+400% −41%	[124]

LAM = Laminarin, FUC = Fucoidan; ^a^ TBARS, thiobarbituric acid-reactive substances; ^b^ FRAP, ferric reducing ability of plasma; ^c^ TAS, total antioxidant status; ^d^ DPPH, 2,2-diphenyl-1-picrylhydrazyl assay; ^e^ T-AOC, total antioxidant capacity; ^f^ SOD, superoxide dismutase; ^g^ CAT, catalase; ^h^ MDA, malondialdehyde; ^i^ GSH, glutathione.

**Table 8:** Potentially toxic trace element concentrations in seaweeds (mg/kg DM).

Trace Element	Brown Seaweed ^1^	Green Seaweed ^2^	Red Seaweed ^3^	Feed ^4^	Feed Ingredient ^4^	Ref.
Cadmium	0.05–8	0.03–4	0.04–3.8	0.5 (1 *)	1	[19,21,39,133]
Mercury	<0.005–0.16	0.005–0.07	<0.005–0.03	0.1 (0.2 *)	0.1	[19,21,39,133]
Lead	0.01–7	0.05–7	0.01–19	5	10	[19,21,133]
Arsenic	8–120	0.8–18	1–50	2 (10 *)	40	[19,21,39,133]
Inorganic arsenic	0.03–7.7	0.2–0.4	0.03–0.6	–	–	[21,133]

^1^*Brown seaweed: Alaria esculenta, Ascophyllum nodosum, Fucus spiralis, Fucus vesicolosus, Himanthalia elongate, Laminaria digitata, Lamibaria hyperborea, Laminaria* spp., *Pelvetia canaliculata, Saccharina latissima Sargassum fusiformis, Undaria pinnatififda*; ^2^ Green seaweed: *Cladophora rupestris, Codium adhaerens, Codium vermilara, Enteromorpha intestinalis, Ulva lactuca, Ulva* spp.; ^3^ Red seaweed: *Chondrus crispus, Gigartina* spp., *Gracilaria vericulophylla, Gracilaria* spp., *Palmaria palmata, Polysiphonia lanosa, Porphyra* spp.; ^4^ UE directive 2002/32/EC and amendments; * Fish feed.
